# “Voice is the New Blood”: a discourse analysis of voice AI health-tech start-up websites

**DOI:** 10.3389/fdgth.2025.1568159

**Published:** 2025-05-29

**Authors:** Alden Blatter, Hortense Gallois, Emily Evangelista, Yael Bensoussan, Bridge2AI-Voice Consortium, Yael E. Bensoussan, Jean-Christophe Bélisle-Pipon

**Affiliations:** ^1^ Faculty of Health Sciences, Simon Fraser University, Burnaby, BC, Canada; ^2^ Department of Otolaryngology - Head & Neck Surgery, Morsani College of Medicine, USF Health, Tampa, FL, United States

**Keywords:** voice biomarkers, voice AI, medical AI, start-up, health technology, discourse analysis

## Abstract

**Introduction:**

Voice as a biomarker has emerged as a transformative field in health technology, providing non-invasive, accessible, and cost-effective methods for detecting, diagnosing, and monitoring various conditions. Start-ups are at the forefront of this innovative field, developing and marketing clinical voice AI solutions to a range of healthcare actors and shaping the field's early development. However, there is limited understanding of how start-ups in this field frame their innovations, and address—or overlook—critical socio-ethical, technical, and regulatory challenges in the rapidly evolving field of digital health.

**Methods:**

This study uses discourse analysis to examine the language on the public websites of 25 voice AI health-tech start-ups. Grounded in constitutive discourse analysis, which asserts that discourse both reflects and shapes realities, the study identifies patterns in how these companies describe their identities, technologies, and datasets.

**Results:**

The analysis shows start-ups consistently highlight the efficacy, reliability, and safety of their technologies, positioning them as transformative healthcare solutions. However, descriptions of voice datasets used to train algorithms vary widely and are often absent, reflecting broader gaps in acoustic and ethical standards for voice data collection and insufficient incentives for start-ups to disclose key data details.

**Discussion:**

Start-ups play a crucial role in the research, development, and marketization of voice AI health-tech, prefacing the integration of this new technology into healthcare systems. By publicizing discourse around voice AI technologies at this early stage, start-ups are shaping public perceptions, setting expectations for end-users, and ultimately influencing the implementation of voice AI technologies in healthcare. Their discourse seems to strategically present voice AI health-tech as legitimate by using promissory language typical in the digital health field and showcase the distinctiveness from competitors. This analysis highlights how this double impetus often drives narratives that prioritize innovation over transparency. We conclude that the lack of incentive to share key information about datasets is due to contextual factors that start-ups cannot control, mainly the absence of clear standards and regulatory guidelines for voice data collection. Addressing these complexities is essential to building trust and ensuring responsible integration of voice AI into healthcare systems.

## 1 Introduction

Voice as a biomarker is a nascent but promising field of research, emerging at the intersection of digital technology and healthcare. Propelled by unprecedented advances in artificial intelligence (AI) over the past decade, vocal biomarker research and its various clinical applications hold transformative potential for non-invasive diagnostics, personalized medicine, and early disease detection (1, 2). Voice biomarker research is also the basis of a promising health-tech market, evaluated at $1.9 billion in 2021 and projected to exceed $5 billion by 2028 (3).

However, there are still ethical, legal, social, and technical uncertainties in this new field. While voice data enables convenient, remote, cost-effective data collection (1, 4), public research—conducted by academic, government-funded, and non-profit entities—reports significant challenges likely to impact the future of the field. These challenges include the lack of accessible, high-quality, and ethically sourced voice datasets, as well as the absence of standardized data collection practices (4–6). Start-ups are pioneering innovation in the field, developing and beginning to market health-tech products based on voice biomarker technology (also referred to as *voice AI health-tech*). While start-ups play a critical role in the research, development, and marketization of these promising voice AI tools, the ways they are describing and characterizing voice AI health-tech at this early stage, and how their discourse may affect the field and end-users' perception has not yet been studied.

This study examines the discourse published on the websites of 25 start-ups developing voice AI health-tech products. Using constitutive discourse analysis, we investigate how these start-ups describe themselves and their voice AI technologies and products. Websites are a vital means of communication for start-ups, often representing the first point of contact for investors and customers (7). Website language is an integral part of the start-up's identity-formation process and a critical source of discourse to convey start-up intentions. Start-ups are tasked with carefully selecting their discursive strategies to positively impact perceptions of their organization and innovations, especially in novel fields. The goal of the study is to uncover how these start-ups strategically position themselves within a dynamic and competitive market while navigating challenges such as limited evidence on vocal biomarkers, limited and underdeveloped regulation, high demand for public trust, and uncertain uptake and integration of their products into healthcare systems. Public-facing websites serve as central communication platforms, influencing perceptions among interested parties, including investors, clinicians, and the greater public. By analyzing the discourse on these websites, this study reveals how start-ups strategically frame their innovations to project legitimacy and reliability to potential partners, funders, and end-users. It also explores potential gaps or synergies between private sector messaging and broader societal expectations, including ethical standards and governance needs. Ultimately, this analysis provides insights into emerging market trends and highlights opportunities for developing robust voice data governance frameworks that foster transparency and collaboration between the public and private sectors.

## 2 Materials and methods

This study employs an adapted constitutive discourse analysis, a post-structural method based on the view that reality is a product of social construction (8). In accordance with the 4-step model developed by Potter and Wetherell (9), the constitutive discourse analysis method used in this study consists of sample identification, website scraping, grounded coding, and analysis.

### 2.1 Introducing constitutive discourse analysis: definition and objective

Discourse analysis is an established field dedicated to examining language use as a form of social action. This method and its sub-types provide lenses through which the construction of meaning, identity, and relationships can be analyzed. In this study, constitutive discourse analysis is leveraged to identify and investigate how the discursive patterns of start-ups in voice AI health-tech *constitute* subjectivities of this emerging field more broadly. Originating in the field of social psychology, constitutive discourse analysis is particularly well-suited for meso-level discourse (as opposed to micro-level, e.g., situated conversation analysis, or macro-level discourse, e.g., systemic phenomena) (10). Constitutive discourse analysis has been used in fields thematically related to this study such as marketing research and the sociology of science (11, 12).

**Figure 1:**
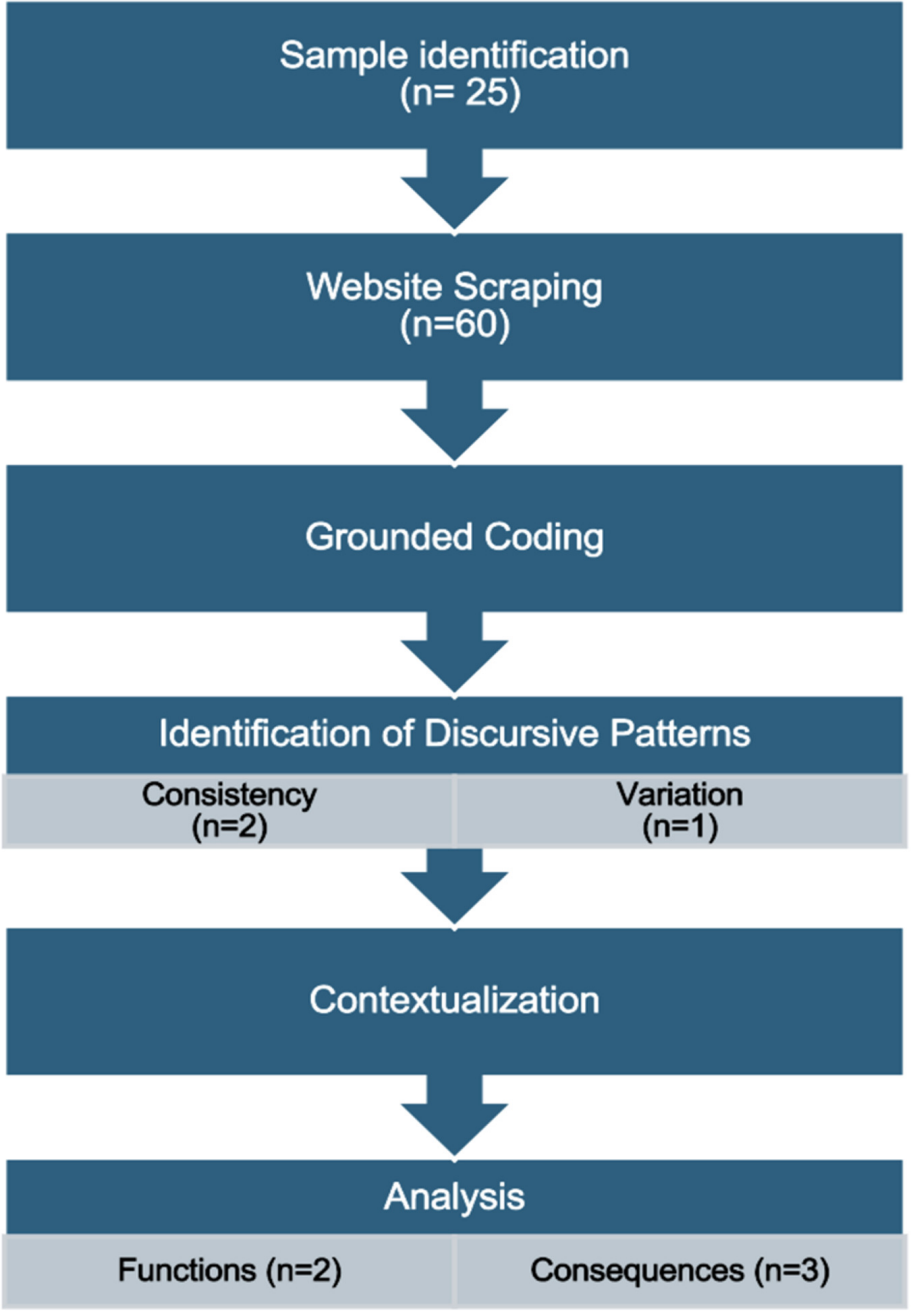
Summary of the constitutive discourse analysis method steps.

**Figure 2:**
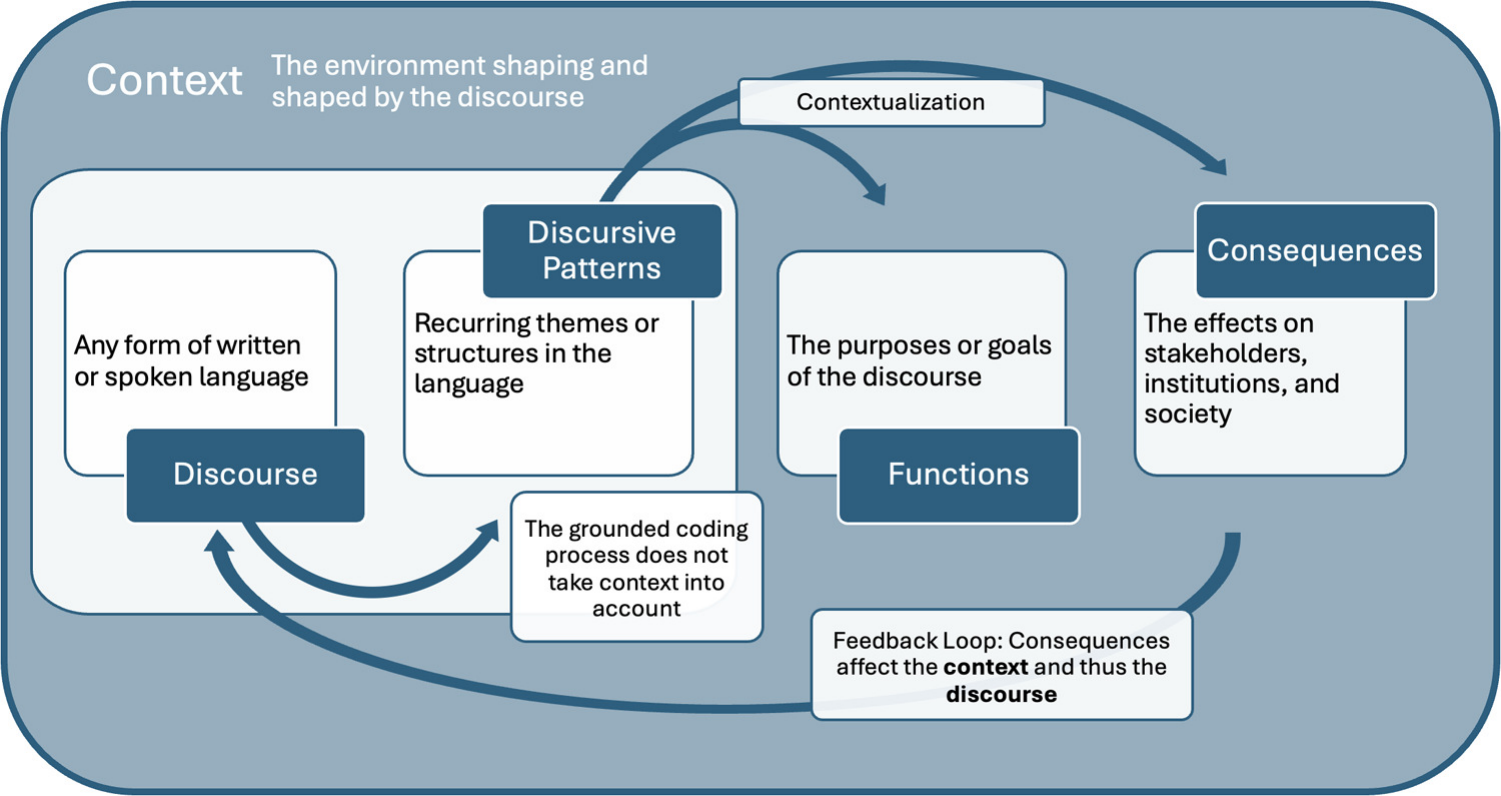
Summary of the analysis phase of the method.

Discourse is defined inclusively as “any form of spoken interaction, formal and informal, and written texts of all kinds” (11). Potter and Wetherell argue that we use discourse for various *functions* (to order, persuade, accuse, etc.), and the linguistic choices they make vary according to the function they are pursuing. Identifying *discursive patterns* is the first step, with the aim being to hypothesize the intended (or unintended) functions of those patterns in their *context*, and finally to consider the *consequences* of those discursive patterns. Figure 1 presents the steps of the adapted constitutive discourse analysis method and Figure 2 illustrates the relationships between the patterns, functions, and consequences in the analysis portion of the method in more detail.

### 2.2 Sample identification

The sample identification process was completed in collaboration with Evangelista et al. (13). Google searches between September 2022 and January 2023 with different combinations of keywords (acoustic biomarkers, vocal biomarkers, voice as a biomarker, acoustic analysis, start-ups, companies, and investments) were used to identify start-ups in the field. Content broadly related to voice AI and voice as a biomarker of health including news articles, press releases, editorials, financial reports, advertisements, and scientific publications was also screened to identify relevant companies (13). To be included in the study, start-ups must have an English-language website and focus on the utilization of vocal or speech analysis to screen, diagnose, monitor, or treat disease. Exclusion criteria can be found in Table 1. Two researchers (EE and HG) identified 24 start-ups that fit these criteria. One start-up (#23) was identified later, before the grounded coding process began, deemed to fit the inclusion criteria, and added to the study, bring the total sample to 25 start-ups.

**Table 1:** Sample identification exclusion criteria.

Criteria	Description
Non-English languages	Start-ups with websites in language other than English were excluded.
Non-human health focused	Start-ups involved in vocal analysis for non-health-related purposes were excluded.
Not related to voice as a biomarker of health	Start-ups that did not use voice as a biomarker of health to predict, monitor, diagnose, or treat disease were excluded.
No website	Start-ups that did not have a public-facing website were excluded.
Not active as of July 2023	An active start-up was defined as a start-up that had a functional website that had been updated in the past 1 year. Due to the dynamic nature of acquisitions in the start-up world, start-ups that have been acquired up to July 1, 2023, were not analyzed independently and were reported as part of the acquiring entity.

**Table 2:** Finalized codebook from grounded analysis.

Code/sub-code	Definition
Artificial intelligence	Mentions of AI-related words and synonyms.
Voice biomarker, voice biometric (and other qualifiers)	Various terms used to describe voice AI technology.
Catchy tropes	Phrases or concepts designed to capture attention or create strong associations with voice AI or related topics.
Comparative descriptors (with existing methods)	Comparisons made between voice AI and traditional or alternative methods in terms of benefits or shortcomings.
Data	Mentions of data (either used to train the technology or data collected from/produced by end-users).
Voice data	Data derived from voice recordings or analysis (including speech, respiratory sounds etc.).
Descriptors	Characteristics or qualities attributed to voice AI products.
Accessibility	Ease of access or use.
Accuracy	Precision or reliability of results.
Cost-effectiveness	Resource efficiency in achieving outcomes.
Efficiency (time)	Speed or time saved through the technology.
Non-invasiveness	Absence of physical intrusion, risks or sense of comfort for the end-user.
Objectivity	Neutrality, lack of bias in outcomes.
Scalability	Potential to expand or adapt to various contexts, media or uses.
Empowerment	Mentions of how voice AI enables users to take control of or improve their health.
Privacy/Security	Considerations related to the protection of data and user confidentiality.
Information on creator	Information about the developers or the start-up.
Information on end-user	Information about the target audience for a voice AI product.
Description of Science/Technology	Explanations of the underlying technology or scientific principles.
Superlative descriptors	Use of terms emphasizing superiority or excellence.
Future	Mentions of potential developments or visions for voice AI.
Becoming a reality	Discussion on moving from concept to widespread clinical use.
Voice AI as new standard	Assertions or implications that voice AI will become the primary or default method in healthcare.
Reference to academic literature	Mentions or citations of scholarly work or studies.
Validation/Certification	References to processes ensuring the reliability or regulatory compliance of voice AI.

### 2.3 Website scraping

Each of the 25 start-up websites was carefully reviewed by two researchers (HG and AB) to select all webpages providing information about the start-ups and their voice or speech-based products, services, and technologies (e.g., webpages labeled Home, About, Technology, Products, Science, FAQ, etc.). Webpages or parts of webpages containing blog posts or referring to external links or documents (e.g., media headlines, academic publications) were not included in this sample as the scope of this study is limited to the discourse produced by the start-ups themselves and published on their website (not an external link). Selected webpages were screen captured between February 8th and March 6th, 2023. For the start-up (#23) that was discovered later (on March 3, 2024), the Wayback Machine was used to access the version of start-up's website that was publicly available in February 2023. Due to variation in website design (the total number of webpages per website), between one and seven webpages were captured per start-up. In total, 60 webpages were selected, captured, and uploaded into the qualitative analysis program NVivo 14 (14).

### 2.4 Grounded coding

Grounded coding is the process of categorizing words, phrases, and paragraphs into themes without a pre-defined coding grid. This “grounding” allows for themes, patterns, and discrepancies to emerge directly from the text. The text is examined from a literal, neutral perspective, without making assumptions based on the context or the researcher's presuppositions about the creator of the text. For example, the code “Becoming a Reality” emerged from the observation of a repeated phrase in the quotes “Extracting medical patterns from voice is becoming a reality” (#1) and “Detecting diseases from the voice? What initially sounds like a future scenario could soon become a reality thanks to modern AI technology” (#2).^1^

An initial coding grid was built by one researcher (AB) during the first round of grounded coding. The researcher (AB) then refined the coding grid, merging, adding, or removing irrelevant codes (see Table 2 for final coding grid). This coding grid was reviewed and validated independently by each member of the research team (HG, JCBP). A second round of coding was then conducted to apply the refined coding grid to all the webpages. Finally, three co-authors (AB, HG, JCBP) reviewed the results to determine the coding process was complete.

## 3 Analysis

### 3.1 Identification of discursive patterns

The first phase of the analysis consists of identifying discursive patterns in the sample. *Discursive patterns* are features of the sampled texts that are either consistent or varied throughout the sample. These patterns can either relate to the content (i.e., topics mentioned, recurring themes, etc.) or the form of the text (i.e., how topics are described; stylistic choices etc.). Discursive patterns emerge naturally from the grounded coding process, as each code is created when a pattern is identified. Some of the identified discursive patterns consist of multiple related codes, while others are a single code. Not all codes that emerged from grounded coded are reported in the results section as discursive patterns, only those for which hypotheses for their function were made in the process of contextualization. Patterns of consistency are expressed with a quantitative element (by presenting the quantity as *n*, and relative weight as a percentage) to give the reader a sense of the degree of consistency of a pattern in the sample. Patterns are qualified as being varied the content is consistent between multiple start-ups, but the form is varied. For example, of the 32% of start-ups that mention the voice datasets used to train their AI technology, only one start-up thoroughly describes their data collection methodology. This outlier start-up thus represents variation in the sample.

### 3.2 Hypothesizing functions and consequences through contextualization

In the second phase of the analysis stage, hypotheses of the functions and consequences of the discursive patterns are raised, and linguistic evidence is provided from the start-up website discourse to verify these hypotheses (9). *Functions* are defined as the underlying purposes of discursive patterns, in other words, the reasons why the sampled start-ups choose the words and language that they do. *Consequences* are broadly defined as the effects discursive patterns have on various actors (e.g., individuals, groups, institutions, etc.) and the society in which the discourse is produced. In this study, hypotheses are made by examining the *context*, defined as the reality or environment in which the discourse is constructed. In practice, this process of *contextualization* was carried out by analyzing the existing literature on the different contextual elements that affect the behavior and discourse of the sampled voice AI health-tech start-ups.

As illustrated in Figure 2, the consequences of the discursive patterns are part of a feedback loop as they are both *constitute* the discourse and context and are *constituted by* the discourse and the broader context. As in biological evolutionary processes, there is reciprocal causation in this relationship, whereby discourse is both constitutive of and constituted by: (a) the actors that produce it (e.g., voice AI start-ups) and the audience the start-up intend to reach (e.g., end-users and investors); (b) the environment in which it is produced (e.g., the broader voice as a biomarker research field and the socio-economic context of modern healthcare systems). Examining this reciprocal causation is in line with other studies that explore how “health discourse is mutually shaping/shaped by current digital environments” (15).

## 4 Results: identification of discursive patterns

This section presents the discursive patterns identified in the text of the voice AI health-tech start-up websites. Three main patterns emerged during the grounded coding and analysis phases (see Table 3): a pattern of thematic consistency in the descriptive words used to qualify voice AI technology (A); a pattern of stylistic consistency in the use of comparative and superlative to qualify voice AI and/or the start-up (B); and a pattern of variation in the quantitative and/or qualitative descriptors used to qualify voice datasets used in the development and training of a voice AI technology (C). While the first two patterns relate to the way start-ups tend to qualify their technology, their organization, or their team, the last pattern relates to the way start-ups describe (or do not describe) their training voice datasets.

**Table 3:** Summary of discursive patterns.

Pattern	Key features	Findings
Pattern A: thematic consistency of descriptors	Recurring use of specific terms to describe technology. Categorized into themes: efficacy, reliability, and safety.	• Efficacy: terms like accessibility, cost-effectiveness, scalability, and time-efficiency (e.g., “real-time,” “device agnostic”). • Reliability: emphasis on accuracy and objectivity (e.g., “92% accuracy,” “quantifiable”). • Safety: focus on data security, non-invasiveness, and user control.
Pattern B: stylistic consistency of comparative and superlative language	Use of comparative terms to position technology as superior to existing methods and superlative terms to claim leadership within the sector.	• Comparative: highlights advantages over non-voice AI methods (e.g., “easier, cheaper, faster”). • Superlative: claims of being “world-leading,” “first-of-its-kind,” or “best-in-class.”
Pattern C: variation in descriptors for voice datasets	Differences in how voice datasets for AI training are described or omitted. Some focus on diversity, robustness, or size, while many omit details entirely.	• 68% of start-ups do not describe their training data. • Of those that do, terms like “diverse” and “unbiased” are used, with some providing detailed quantitative descriptions (e.g., “43,000 audio sessions”). • Data bias and methodology are addressed by only one start-up.

### 4.1 Pattern A: thematic consistency of descriptors

The first discursive pattern that arose from the grounded coding process was the recurrence of similar themes. Specifically, most websites use similar descriptive language (hereafter “descriptors”) to characterize their voice AI technology. The most consistent descriptors are categorized in nine themes: accessibility, time-efficiency, cost-effectiveness, scalability, accuracy, objectivity, data security/privacy, non-invasiveness, and control. Although each of these descriptors (and their identified synonyms) hold a specific meaning, these terms either tend to convey efficacy (i.e., in terms of gains of time, resources, or ease to access/use for the end-user); reliability (i.e., in terms of accuracy and objectivity); or safety (i.e., the absence of physical risks for the end-users).

#### 4.1.1 Efficacy (accessibility, time-efficiency, cost-effectiveness, and scalability)

Descriptors related to the ease of access/use and efficiency of voice AI products are the most consistent pattern identified in the sample, used by 19 out of 25 start-ups (76%). Examples of descriptors related to *accessibility* include “easy to use,” “usable for anyone, anywhere, anytime,” “compatible with mobile devices or device agnostic,” “simple,” “seamless,” “age/elderly friendly,” and “convenient.” Terms like “democratizing” are also used to emphasize the ease of access: “accelerate innovation and personalization in healthcare by democratizing access to advanced technology solutions and real-world data to deliver long impact results to patients” (#18). *Efficiency* is most often used to refer to time. “Fast results,” “real-time,” and “instantly” are examples of descriptors that were coded under this theme, reflecting the speed of these novel AI-based technologies to analyze voice samples. The concept of time-efficiency is also expressed by quantifying the duration of voice samples necessary for technology to analyze and deliver results. For example, one company states “6–30 s for voice health detection” (#20), while another states “Earlier Alzheimer's detection anywhere in 10 min” (#15).

*Cost-effectiveness* is a descriptor used by 60% (*n* = 15) of start-ups. It is both referenced as an absolute; “hardware free, extremely low cost, scalable, remote or local” (#7); and in relation to existing diagnostic or monitoring tools/technologies; “[#6] allows cost-effective, non-invasive monitoring for clinical deterioration and patient monitoring – detecting issues earlier and preventing readmission.” Cost-effectiveness is sometimes used in relation to specific beneficiaries such as patients, clinicians/health care providers, or insurance companies. For example, under the subtitle “patients”, one start-up states that they their technology can “save time, anguish, and money by reducing hospital visits,” and below, under the subtitle “health care providers,” it states, “Detect flare ups earlier and administer treatment to prevent deterioration, improved coordination and delivery of care, save costs of hospitalization” (#12). Another start-up (#22) states that “our voice biomarker algorithm and care coordination platform lowers the cost of care by reducing 9%–13% of unnecessary hospitalizations,” in the section of website headed “Insurance Companies.” Cost-effectiveness is also used more broadly in one case, in which a start-up states that an advantage of their product is “reduced expenses and increased financial margins throughout the continuum of care” (#9), seemingly relating cost benefits for all actors in the healthcare system.

*Scalability* is the last consistent descriptors in this category (*n* = 11; 44%). The sampled start-ups use the terms “scalable,” “at scale,” and “replicable,” seemingly to express the potential of their technology to expand, although the term is not explicitly defined by any of the start-ups. One start-up states: “Scalable: mass deployable sensor, only standard microphone needed” (#2). Another start-up claims “Truly borderless wellness. Language-agnostic and device-agnostic, [#6] powers global solutions,” showing that their technology functions at the global scale.

#### 4.1.2 Reliability (accuracy and objectivity)

*Accuracy* is used by half of the sampled start-ups to describe their technology (*n* = 13; 52%). Accuracy is referred to in absolute terms, in relative terms compared to existing diagnostics, and is often quantified with percentages. For example, one start-up claims that “Voice AI detects even slight symptoms, signiﬁcantly increasing the chances for early detection. With COVID-19 or Parkinson's we reach up to 92% accuracy” (#2). It is unclear what it means to reach “up to” 92% accuracy.

Over a third of start-ups (36%, *n* = 9) used terms related to *objectivity* to qualify their technology, including two start-ups that used the descriptor “quantifiable.” For example, two start-ups emphasize the objective nature of technology as compared to traditional (human-based) diagnostic to convey the idea of reliability and lack of bias in diagnostic tools. This can be seen in these two quotes: “Majority of diagnostic and treatment decisions derive from subjective, secondary, biased and spontaneous reporting data. Given the heterogeneity and evolving nature of practice, novel approaches are required that actively learn from each patient and provide evidence-based metrics, so as to reduce treatment resistance” (#18); “We're bringing healthcare into the digital era using voice AI, replacing subjective measurements with objective, actionable data” (#6).

#### 4.1.3 Safety (data security/privacy, non-invasiveness, and control)

Start-ups consistently mentioned the *security* measures taken to protect the user's voice data collected by their technology (*n* = 11; 44%). Six start-ups (24%) do this by qualifying their product as compliant with American (HIPAA) or European (GDPR) health data privacy regulations. Others simply qualify their product's platform as “secure” or “safe.”

Although less consistent than other descriptors, the ease, comfort, and safety of voice AI as a *non-invasive* technology is reported by 20% of the start-ups (*n* = 5). In one case, a start-up compares their technology to existing methods, stating: “Our real-time speech analysis API detects diseases and clinical conditions earlier and less invasively than traditional methods.” Non-invasiveness is also used to generate the idea of comfort and ease for the end-user. For example, non-invasiveness is used in reference to the data collection procedure: “non-invasive audio collection for comfortable, stress-free analysis anytime, anywhere” (#6).

Another theme that emerged refers to generating a sense of *control over one's own health*. Seven start-ups (28%) characterize voice AI technology as a way to empower end-users to manage their own health: “empower patients to diagnose and manage respiratory disease” (#17); “empower people to more effectively manage their health” (#20); “we aim to deliver accessible, affordable, accurate health insights so you can take charge of your health” (#19); “giving you control over your health” (#9). In another example, empowerment is also broadened to caretakers, beyond patients: “this is the only non-invasive, easy to use medical grade, CHF monitoring device that offers patients and caretakers peace of mind and a true sense of control” (#9).

### 4.2 Pattern B: stylistic consistency of comparative and superlative language

A second consistent pattern emerged in the stylistic ways start-ups described themselves. This consistent pattern relates to the use of comparative and superlative language when describing either the technology or the start-up itself. This pattern conveys a sense of superiority, either in absolute terms (i.e., generalization of excellence detached from any comparison point) or relative to existing methods not reliant on voice AI or other voice AI health-tech tools.

#### 4.2.1 Comparative descriptors: superiority over existing (non-voice AI) methods

Most start-ups (64%; *n* = 16) use comparative descriptors to qualify their technology favorably to existing disease detection, diagnostic, or monitoring tools. Relative terms such as easier, cheaper, faster are frequently used. For example, one start-up states: “Our vocal biomarker technology is more accurate and models more data points than existing screening methods. [#6's] vocal signatures, AI, and machine learning detect mood and disease states before presentation of observable symptoms and ahead of traditional clinical screening” (#6).

A third of the start-ups (32%; *n* = 8) take their comparisons with existing tools further by claiming that voice AI will become the new standard of healthcare. Phrases such as “Voice AI is the future, and [#6] helps partners stay ahead of the curve,” (#6) and “We are surfing the wave of innovation with those who can keep up with the speed” (#24) exemplify this discourse. One company states at the top of their home page that “Voice is the new blood,” (#1) seemingly alluding to voice replacing blood as the standard sample or source of health data for medical testing. Other companies state that they are “unlocking a new paradigm of patient care,” (#3) “giving voice to a new standard of care,” (#10) “creat[ing] the next generation of care,” (#18) or providing “the new standard for assessing wellness” (#6). Another start-up states as the headline of their homepage “Today, there is no objective and scalable measure for the severity of anxiety and depression,” with the sub-header “We're solving that problem” (#10). These statements characterize the impact their technology will have on healthcare as inevitable and transformative.

#### 4.2.2 Superlative descriptors: superiority among voice AI health-tech

Superlative descriptors are also used somewhat consistently (28%; *n* = 7) in the sample to qualify either the technology marketed on the website, the company and its team, or both. These superlatives include terms such as “best,” “most innovative,” and “most cutting-edge.” Start-ups qualify their company or their technology as “world-leading,” “world's most advanced,” “pioneering,” “first-of-its-kind,” or “best-in-class.” For instance, start-up 6 describes its technology and developers as “Best-in-class voice AI SaaS, brought to you by industry leaders in speech and language technology,” and “First-of-its-kind patented vocal biomarker technology.” The same start-up also states that they have been “awarded more patents than any existing speech AI company” (#6). Another start-up claims that they have the “Most Cutting-Edge Voice AI Technology” (#23). Finally, some start-ups showcase unique validation of their technology, for example, having the “only patents specific to screening for disease using voice AI” (#6) or the “Only Clinically Validated Vital Sign for Mental Health” (#10).

### 4.3 Pattern C: varied amount of information about training data

A discursive pattern of variation emerged from the analysis of the ways the sampled start-ups qualify the voice datasets used to train or develop their voice AI technologies. Voice data is mentioned in the sample as one of two categories: (1) voice data collected from the end-user's use of the voice AI product; or (2) voice datasets (and possibly associated health information) that are used to train the AI technology. The first category is often described in relation to privacy and security (see above in the “Safety” subsection of Pattern A), but the second category of data is less consistently described or mentioned. Only eight start-ups in the sample (32%) mention their training voice datasets on their websites, most frequently to characterize them as “diverse,” either explicitly or indirectly. Notably, the other 17 start-ups (68%) did not mention or qualify the data used to train their technology anywhere on their websites.

Four start-ups provided a few numerical statistics to characterize their data collection such as “25 countries, 190 sites, 24 languages, 140,000 recordings,” (#16) “85,000+ individual research subjects, 1.2 million + voice samples” (#20), or “almost 400 million sound samples, all of them collected in the real world, across almost every country and every socio-demographic segment” (#13). Others characterized their data/datasets as diverse (*n* = 3), unbiased (*n* = 1), robust (*n* = 2), or “world's most comprehensive” (*n* = 1). Only one start-up explained the importance of diverse data, stating “AI models identify patterns based on training data. Bias can find its way into AI models if the training sample is skewed. For example, if a group of people were over- or underrepresented in the sample, or if their data was collected during unusual circumstances (such as during a pandemic)” (#8). This same start-up also reported that “Our researchers are continuously verifying that our models are not biased based on demographics, environmental conditions, timing, or other factors” (#8).

Finally, one start-up provided a thorough quantitative and qualitative description of their training voice dataset, acting as an outlier among the start-ups.

Patients were diagnosed and recruited by psychiatrists from six different mental health hospitals across the [country (de-identified)], following DSM-5 standards. Patients were given an H5 miniprogram for the voice sample collection, and the collection process was carefully designed, covering long vowels, number counting, rainbow passages, speech under cognitive load, open questions etc. Our mental health dataset [de-identified] now contains more than 43,000 audio sessions, collected from depression patients, anxiety patients, non-depression non-anxiety people etc., and it's by far the biggest audio dataset from DSM-5 diagnosed patients (#23).

This description includes details on the start-ups' data collection methodology, including the location and number of collection sites, the type of voice samples collected, and disease category of participants. Overall, the most notable discursive pattern related to data in this sample was the pattern of omission, with 68% of the companies not mentioning their AI training data.

## 5 Discussion: functions and consequences of the voice AI health-tech start-up discourse

This adapted constitutive discourse analysis method relies on contextualization to hypothesize the functions of the discursive patterns identified in the results (see Figure 2). While each start-up is operating in its own local, situated context, the start-ups in this sample have a number of contextual elements in common. These commonalities are examined to contextualize the sampled start-up website discourse and hypothesize why they are using the language they are. The first common contextual element is that all 25 start-ups are operating in countries whose healthcare systems are in the process of digitalization. Second, these start-ups seem to be targeting a common audience with their websites, which includes diverse range of actors. Third, these start-ups are part of the “digital health” sector and aim to project legitimacy and create value for their innovative products within this space. Fourth, voice and speech biomarker research (in both the public and private sectors) is still quite new, and standards for datasets in this field are unclear or undetermined. Fifth, the private sector is not incentivized to be transparent about their training datasets. Two hypotheses of functions emerge from the contextualization process: (1) consistent patterns A and B (thematic and stylistic descriptors) serve to simultaneously legitimize the voice AI health-tech as part of the larger digital health sector and distinguish start-ups and their products from their competitors within the smaller voice AI health-tech field; (2) pattern of variation C (training data) is due to a lack of standards in generating voice datasets for health and incentives for private industry to be transparent about their data. These hypotheses are introduced through the context and then linguistic evidence from the results is presented to validate them (see Table 3 for a summary of the discursive patterns and Table 4 for a summary of the functions and consequences).

**Table 4:** Summary of functions and consequences.

Category	Key points	Implications
Contextual factors	• Common external contexts include digitalization of healthcare, fragile start-up business models, and the nascent state of voice AI health-tech. • Challenges include aging populations, healthcare professional shortages, and high costs. • Start-ups must appeal to diverse audiences: end-users (patients, clinicians), investors, insurers, and regulatory bodies.	• Drives start-ups to strategically use website discourse to establish legitimacy in the digital health sector while emphasizing distinctiveness in the voice AI health-tech field. • Highlights the complex audience landscape, requiring tailored communication for different stakeholders.
Pattern A & B: legitimate distinctiveness	• Start-ups use legitimacy discourses to align with broader digital health narratives (e.g., efficiency, accuracy, empowerment). • Use distinctiveness discourses to differentiate themselves through superlative language (e.g., “best-in-class,” “world-leading”).	• Legitimacy discourses promote voice AI health-tech as fitting into the digital health sector, ensuring trust and investment. • Superlative language creates differentiation but may be secondary to legitimacy in this nascent field. • Alignment with broader promissory discourses (quantification, connectivity, instantaneity) helps attract stakeholders.
Pattern C: opacity in data descriptions	• Majority of start-ups (68%) do not describe training datasets, reflecting a consistent omission or variation in transparency. • Issues include lack of data availability, absence of standards, and opaque regulatory environments. • High-quality and diverse training datasets are critical for reliability but expensive and complicated to obtain.	• Lack of transparency risks compromising stakeholder trust and algorithmic reliability. • Creates uncertainty for clinicians, investors, and patients about the quality of the products. • Reflects broader challenges in the nascent field, where regulations and standards have yet to be established.
Consequences: patterns A & B	• Promissory framing reconfigures healthcare relationships (e.g., doctor-patient interaction) and knowledge systems (e.g., turning qualitative health assessments into quantitative metrics). • Digital health discourses reshape healthcare tasks, emphasizing patient empowerment and self-management.	• Can lead to overconfidence in untested tools, potentially misconfiguring trust relationships. • Reconfigurations, while often beneficial, may result in inequities if digital health solutions are not accessible or misused. • Raises ethical concerns about presenting a one-sided narrative of the technology without addressing risks or limitations.
Consequences: pattern C	• Lack of transparency amplifies risks of algorithmic evidence (e.g., bias, unreliability, inconsistency across populations). • Demonstrates parallels with failures in the health-tech sector (e.g., Theranos), highlighting the risks of “stealth research.”	• Training data opacity undermines trust and risks algorithmic bias. • Transparent standards for dataset quality, diversity, and biases are critical for mitigating risks and ensuring adoption. • Public trust in the broader field of voice biomarkers may suffer if opacity leads to failures or scandals.
Public trust and recommendations	• Public trust can be undermined by exaggerated promises and opaque practices. • Excessive trust in AI-based tools can lead to blind faith in flawed systems, while insufficient trust may hinder adoption. • Transparency is vital for building digital trust, aligning with frameworks such as the World Economic Forum's Digital Trust Framework (cybersecurity, safety, transparency, etc.).	• Promotes the need for transparent, validated communication about datasets and technology. • Advocates for avoiding the pitfalls of stealth research (e.g., Theranos). • Recommends adopting established guidelines for transparency and public trust until regulations are formalized.

Three hypothesized consequences emerge from this process. The first is that the consistent descriptors of patterns A and B describe significant reconfigurations of healthcare as objectively positive for a variety of actors, but in practice these reconfigurations are unpredictable and could also have negative impacts on patient outcomes or doctor-patient relationships. The second consequence is that pattern C, lack of information about training data, could amplify the inherent risks of using algorithmic evidence for health applications. Third, it is possible that patterns A, B, and C could negatively affect public trust in voice AI health-tech while this field is still new and holds much potential.

### 5.1 Pattern A and B: strategic marketing in digital health and unpredictable healthcare reconfigurations

#### 5.1.1 Elements contextualizing patterns A and B

##### 5.1.1.1 Digitalization of healthcare systems

The start-ups identified for the study are all based in Organisation for Economic Cooperation and Development (OECD) member or key partner countries, whose healthcare systems face a similar set of complex and pressing issues: aging populations, shortages of healthcare professionals, and unsustainably high costs for both patients and institutions, among others (16). Digitalization is seen as a way to use technology to solve some of these issues, and healthcare systems in OECD countries have digitalized to varying degrees over the past decades (17). Large and small actors (including start-ups) in the private sector are most often the innovators that leverage technology to produce and market digital tools intended to address the deficiencies of modern healthcare systems.

##### 5.1.1.2 Voice AI health-tech as an emerging market within digital health

Digital health can be defined as knowledge and practice associated with the development and use of digital technologies to improve health, encompassing subfields such as telemedicine or telehealth, mHealth, and algorithmic medicine (18, 19). The products being marketed by the voice AI health-tech start-ups in our sample fit this definition and into these sub-fields, as they use mobile phones (mHealth) to diagnose or monitor diseases or conditions remotely (telemedicine) using AI (algorithmic medicine). Digital health is a burgeoning market that has grown exponentially in the past decade, largely because of government initiatives promoting it to transform healthcare (20). Digital health was valued at $240.9 billion in 2023 and projected to grow at a compound annual growth of 21.9% from 2024 to 2030 (21). Start-ups have become key players in this sector, leveraging the unprecedented technological advancements of the last decade to produce digital health solutions that aim to shift the way we provide care (22).

Voice AI health-tech is a new field within the digital health sector, and the sampled start-ups face the challenge of creating a new market for their innovative voice AI health-tech products. Voice AI health-tech is a promising innovation that could benefit patients, clinicians, and other actors in the healthcare system, but these start-ups must convince these actors, as well as investors, of the value of this innovation before they can market their product effectively. Two additional factors present in the voice AI health-tech field potentially increase reluctance among investors and other interested parties: limited availability of information about firms because of their newness and the lack of actual results firms have to prove the value of their products (23). These start-ups thus must strategically use their website, as their public platform, to justify the legitimacy, distinctiveness, and value of their innovative products.

##### 5.1.1.3 Addressing a diverse audience

The type of discourse used by start-ups on their websites is heavily influenced by their intended audience. A key audience of these websites are the end-users of the product, or “consumer target.” In the case of voice AI health-tech, the consumer target includes a diverse set of actors within the sensitive and fragmented environment of healthcare (i.e., patients, clinicians, health administrators and others within the healthcare system). Each have varying needs, priorities, and literacy levels, and start-ups must carefully tailor their discourse to account for these discrepancies (24). For instance, the information provided on the website must simultaneously be clinical enough to convince clinicians of its validity and simple enough to be accessible to patients in the general public. The audience may also vary depending on the type of product market (e.g., FDA approved medical device for clinical use is distinct from a direct-to-consumer “wellness app”).

Beyond the consumer target (or end-users), the website discourse must also be oriented toward marketing the start-up itself as a valuable investment, beyond their products. Investors are a key target audience of these websites, as the start-up business model relies on external funding until the product is ready to go to market. These investors are typically from the private sector (e.g., venture capital firms, larger technology firms, pharmaceutical companies, etc.), but start-ups can also access funding through public institutions such as the NIH and the National Science Foundation (NSF) through grants and other sources.

Another important part of the audience are public and private insurance payers. Most health-tech products are not intended to be paid out of pockets by end-users, meaning that the traditional rules of supply and demand do not apply in the process of commercialization (25). For example, a voice AI health app may require a prescription from a healthcare professional in order to be paid for by third actor (decision maker in the healthcare system, third-party payer, etc.), which will influence the end-user's (i.e., the patient) decision to buy the product or not (25). As such, the marketing process involves a complex audience because a number of different actors are involved in the acquisition and use of health technologies.

#### 5.1.2 Function: strategic marketing leveraging legitimate distinctiveness

The context of digital health and start-up strategies for creating value in new markets, we hypothesize that the function of the consistent thematic and stylistic use of descriptors identified in the results as patterns A and B is a marketing strategy called “legitimate distinctiveness” (26). Legitimate distinctiveness can be a powerful way to establish a space for an innovative product in an existing market. Innovation is a complex process which motivates social change in a market, but to catalyze that change entrepreneurs must convince investors and other interested parties that their innovation is valuable (26, 27). Entrepreneurs employ a diverse set of discursive strategies to justify the value of their innovation and attain investor support. Legitimate distinctiveness is one such strategy, which aims to convince investors that the company and the product(s) fit into the existing market and simultaneously stand out within it (23). Legitimate distinctiveness assumes that investors first evaluate a start-up or other firm broadly within a relevant category (a market or industry), assessing whether the start-up is a legitimate member of that category. Legitimacy, in this case, is defined as “generalized perception or assumption that the actions of an entity are desirable, proper, or appropriate within some socially constructed system of norms, values, beliefs, and definitions” (28). Investors then make within-category distinctions, assessing distinctiveness from other rival firms (23).

According to this strategy, voice AI start-ups, who are developing an innovative, novel product, seek legitimacy by aligning themselves to fit within the more established digital health sector. The types of descriptors used to qualify the health-tech, the comparisons to existing methods of care, and the “new standard of healthcare” and “empowerment to manage health” discourses are all common in the broader digital health discourse (29). Voice AI health-tech start-ups thus fit in and are legitimized by echoing these promises. Also present is discourse that differentiates start-ups and their voice AI health-tech from the rival firms and their technologies using superlative language, signaling distinctiveness within the field.

##### 5.1.2.1 To legitimize: reliance on promissory discourses of digital health

Marent and Henwood (29) and Pickersgill (30) find that promissory discourses are common in the presentation of digital health technologies as they are with other types of innovative products (29, 30). Quantification, connectivity, and instantaneity are three promised benefits of digital health technologies—ways which technology is expected to transform healthcare—typically laid out in such discourses (29). Quantification refers to the transformation of patient health conditions into data points. Connectivity refers to the possibility of accessing medical services anywhere, at any time. Instantaneity refers to the self-tracking and self-management of disease through digital technologies that monitor patients and prompt them in real-time (29). These three digital health discourses match the descriptors found in the sampled voice AI health-tech website discourse. Quantification aligns with objectivity, accuracy, and scalability. A quote from start-up 3 exemplifies quantification in the sample: “Speech analytics provide objective, repeatable metrics—unlocking a new paradigm of patient care.” In this phrase, speech analytics technology positively transforming patient care through quantification, which is referred to as “objective, repeatable metrics.” Connectivity aligns with accessibility, as these products can be used anywhere, and results can be transferred to a clinician through the internet. Instantaneity aligns with time effectiveness and control. Results from voice AI technologies are delivered “instantly” or in “real-time,” and start-ups highlight that this aspect of the product “empowers” patients, putting them in control of their own health.

Marent and Henwood (18) addresses the “utilitarian argument” and the “empowerment argument” often put forth by digital health companies, both of which also align with promises of better care (Pattern B) and empowered patients (Pattern A, subpoint c) control) (18). The utilitarian argument claims that digital health technologies increase the efficiency, effectiveness, and quality of health services, which the sampled start-ups echo in their use of time efficiency, cost effectiveness, and accuracy. The empowerment argument claims that digital health technologies give patients (and the public more generally) ways to manage their health by being able to access their personal health data and receive timely feedback accordingly. This idea of patient empowerment was also common in the sampled start-up discourse, for example, in this quote: “We aim to deliver accessible, affordable, accurate health insights so you can take charge of your health” (#19). Van Dijck and Poell also identify a common discursive regime among digital health platforms in promising to “transform medicine into personalized health care with the intention to serve the public good,” which relates to the pattern we identified in the sample characterizing voice AI health-tech products as a new standard of care (31). The consequences of these promissory discourses commonly found in the digital health sector will be discussed further in the consequences section below.

##### 5.1.2.2 To distinguish: use of superlatives

The distinctiveness aspect of the legitimate distinctiveness marketing strategy refers to discursive ways firms distinguish themselves from their competitors and thus stand out to stakeholders. Of the start-ups in the sample, 27% (*n* = 7) used some form of superlative to describe their product or company. Start-ups in our sample do this by using superlative descriptors to qualify their product or company or by claiming they have the most validation or are the only start-up to have a certain kind of validation.

We hypothesize that the distinctiveness discourse is not as prevalent as the legitimacy discourse for a few reasons. The first is that the sample of start-ups is relatively diverse in terms of disease cohort and end-user, making these companies/products distinctive from each other. Second, voice AI health-tech is a new market with most of the sampled start-ups being less than 10 years old. Because this sector represents a new, relatively untested market, convincing investors of the legitimacy of the company and product may be more important than standing out from the relatively small field of competitors. As the voice AI health-tech market grows, it is possible that firms begin to distinguish themselves more.

#### 5.1.3 Consequence: unpredictable reconfigurations of healthcare

Marent and Henwood (29) frame quantification as a reconfiguration of knowledge, challenging the conception of health data as neutral and objective by examining how quantifying apparatuses in medicine produce phenomena. Algorithmic technologies are necessarily quantifying, turning human health into representative numerical data. Quantified health data representing the human voice is a reconfiguration of the aural recognition of health that human doctors use to diagnose or monitor conditions. This reconfiguration is not neutral or objective, as claimed by many of the sampled start-ups, it is based on decisions and processes made when data is produced, categorized, and reported (29). Other critical scholars echo this, arguing that quantified health data is necessarily produced through reductive means that affect how the human body is rendered (32). We find that this critique is relevant to the voice AI health-tech start-up discourse, and that the quantification of health data is not as objective or inherently positive as the sampled start-ups characterize it. One example in the sample is the claim by start-up #2 that their technology is “up to 92% accurate.” This claim could mislead users to think that the technology is 92% accurate in any circumstance as there is no context explaining what that percentage means, what clinical endpoint it compares to, or in what population that level of accuracy was attained.

Like quantification, the connectivity and instantaneity provided by digital health technology reconfigures relationships and control within the healthcare system. Different interaction environments, for example, prompts related to a health condition that pop up as a phone notification instead of being reported and explained by a doctor face-to-face, reconfigure the connection one has to their doctor and their health (29). This can have consequences if a patient has a low level of digital competence, misunderstands the prompt, and acts according to that misunderstood information. Additionally, having health conditions constantly monitored by devices and reported through prompts changes how health tasks are distributed. Patients' routinized, intuitive ways of acting are reconfigured by instant and continuous health notifications, and the relationship with their human doctor is affected (29). In sum, the quantification, connectivity, and instantaneity of voice AI health-tech and digital health technologies more generally reconfigure different aspects of healthcare. Reconfigurations are not necessarily negative, and it is commonly argued that healthcare systems need to be reconfigured considering their diminishing capacity to treat an increasing number of patients, but employing consistently promissory, positive characterizations of a technology without addressing any of its challenges or negative implications paints an inaccurate picture of this technology. However, it is important to note that start-ups operate in a competitive space and must convince investors and other actors of the value of their product to survive. In this context, start-ups have little choice but to follow the marketing strategies typical of the digital health sector to maintain their legitimacy.

### 5.2 Pattern C: opacity by necessity and algorithmic risk

#### 5.2.1 Elements contextualizing pattern C

##### 5.2.1.1 Lack of standards for voice data generation and regulatory uncertainties

In the public sector, a key barrier to voice biomarker research is the lack of accessible, large-scale, diverse voice and speech datasets (1, 33). The lack of technical and ethical standards for collecting voice and speech data for health purposes poses another barrier (1, 13, 34). This lack of standards holds back research by limiting the interoperability of data. For example, there is no standard sampling rate and signal to noise ratio for voice and speech datasets for health, even though it has been found that a minimum rate and ratio are necessary for accurate analysis and utility (33, 35). Regarding the private sector, Evangelista et al. (13) finds that there is a lack of standardization in voice data recording methods and data storage among voice AI health-tech start-ups (13).

Voice AI health-tech start-ups need to access or generate voice datasets to train their AI-based technology, just as researchers in the public sector must access or generate datasets to validate their hypotheses. Thus, the issues of data access and data standards in public research affect start-ups as well. Additionally, the complexities of regulatory frameworks governing voice data, such as HIPAA in the United States and the GDPR in the European Union, often lead institutions to classify voice and speech as identifiable biometric data. This classification necessitates stringent protection and control over access to voice datasets to ensure compliance, even when the actual risks to data protection may vary. These stringent regulations can lead institutions to implement rigorous controls over voice data to ensure compliance, sometimes resulting in limited access to such datasets for research and development purposes.

##### 5.2.1.2 Lack of incentives for transparency in the private sector

While the challenges surrounding datasets are shared between the public and private sectors in the voice biomarker field, incentives and norms surrounding publication and transparency differ. In public research, for a study to be published in a peer-reviewed journal, the dataset, including the method by which it was collected, must be thoroughly reported. In the private sector, where profit replaces knowledge production as the key motivator, transparency and the publication of peer-reviewed research are not incentivized. The term “stealth research” was coined in a 2015 article about Theranos and other biomedical innovators to refer to their strategy of keeping the science behind their technology a secret and avoiding publication of peer-reviewed research (36). Start-ups are not incentivized to publish, as it has been found not to impact a company's valuation in any way, and it takes time and limited resources (37). Reticence to publish and thus publicize information about technology and data in the private sector can be seen as prioritizing innovation over transparency. Stealth research and the lack of transparency that comes with it create challenges for actors in the healthcare systems to understand the clinical robustness of claims made by digital health companies, especially because these companies tend to have limited regulatory filings, clinical trials, and publicly available data (20).

#### 5.2.2 Function of pattern C: opacity by necessity

We hypothesize that there is a pattern of variation regarding the amount of information start-ups published about training datasets (pattern C) because there is a lack of standards for such datasets in this novel field, and due to this lack of standards, start-ups face more risks than benefits in being transparent about their datasets. Without established standards for voice data collection and voice datasets for health more generally, start-ups have no baseline with which to compare their data collection practices or datasets. In this context, publishing detailed information about their dataset could open start-ups up to criticism or competitive differentiation. Moreover, there are no laws or regulations that force private sector actors such as start-ups to be transparent about such information. In this environment, it is a risk for start-ups to report information about their datasets. Regarding benefits, there is no precedent for transparency leading to higher investment or other material advantages in the private sector of the digital health field. The lack of benefits is exemplified by the fact that investors have historically not incentivized transparency via peer-reviewed publication in digital health (37). Opacity about training datasets thus seems to be a systemic problem related to the lack of data standards in the field, and the increased risks and lack of benefits for health-tech start-ups to be transparent.

The sampled start-ups that did mention their training datasets generally did so to make their dataset seem extensive and high-quality. This can be seen in the four start-ups that used statistics to quantify and qualify their data, claiming they have, for example, millions of samples from “almost every country and every socio-demographic segment” (#13). There is no way to validate these claims without more information about the data collection process, but at face value the numbers sound impressive, making them a potentially useful marketing tool.

It is unclear why one start-up published a thorough description of their dataset and data collection methodology, but there are few factors that may be at play. The outlier start-up is unique in that it is the only one from this country in the sample. The regulatory environment and norms around transparency may be different in this country, although we could not verify this in our research.

#### 5.2.3 Consequence: algorithmic risk amplified by data opacity

We hypothesize that opacity about training dataset amplifies the already significant risks that come with using algorithms to monitor, diagnose, and predict disease. Although the term algorithmic risk sounds like it centers the quality of the algorithm, it is the data being analyzed by the algorithm that is arguably more important. The quality and quantity of data used to train an AI technology is the most important factor in its reliability (38). A dataset of insufficient quantity and/or quality can introduce various representation biases (39). Sample bias is one example, occuring when there are differences in training and test dataset population distributions (40). Algorithmic models based on correlation work well when the target population is similar to the training data, but when in a dynamic clinical environment with a heterogeneous population a model that worked in one setting may fail in another (41).

Morley and Floridi (42) present a framework of concerns (based on (43) related to algorithmic use in health care, which illustrate the possible consequences of insufficient training data for AI health-tech. Concerns related to the algorithmic evidence (defined as the output or findings derived from computational processes used to analyze or interpret data) being inconclusive (probabilistic and not infallible), inscrutable (minimal oversight of the specific input data used to generate the outcome), and misguided (outcomes are only as reliable as the data they are based on) are all possible risks for the sampled voice AI start-up products. Lack of transparency about the data that trained these AI-based products makes their algorithmic evidence especially inscrutable and unknown if it is misguided, thus amplifying the risk of algorithmic error. These concerns can lead to AI-based health-tech harming individual patients, but also negatively impacting trust at the relationship (clinician-patient), group, institutional, and/or societal level (42). The authors offer the example of an algorithm leading to patient safety issues that regulators are unable to address, resulting in a loss of public trust in the technology, the institution providing the technology, and the regulatory body (42).

Liu et al. (44) exemplifies the concerns related to sample bias and misguided evidence through an examination of the use of an AI-based oncology tool called Watson for Oncology (WFO) in China. WFO is a clinical decision-support system for oncology therapy selection that was developed by IBM in the US. It was introduced in China in 2017 and had served more than 10,000 patients there when the study was published in 2018. The study found that treatment consistency was only 65.8% in China compared to 96.4% reported in the US (44). The authors found that the lower treatment consistency in China was due to differences, mainly patient physique and available drugs for treatment, between Western and Chinese contexts. WFO was reportedly trained on test cases with patients at Memorial Sloan Kettering Cancer Center (MSKCC) in New York City. The study concludes that WFO must be trained on a unique medical data repository for China to function consistently in that context. This case illustrates the importance of using training data that matches the population where the AI model will be implemented. Transparency about the diversity of the training dataset can build trust by giving stakeholders an idea of how the start-up has navigated representation biases.

### 5.3 Transversal consequence: considerations for public trust

We hypothesize that public trust in the broader voice biomarker field may potentially be affected as a consequence of the patterns of promissory descriptors and opacity around datasets in the sampled voice AI health-tech start-up website discourse. The promissory discourses in the sample, which, for example, qualify voice AI health-tech as objectively better than existing screening methods, may inspire too much trust in a new, relatively untested AI-based tool. Asan et al. (45) argue that the human-AI trust relationship should be delicately calibrated because a high level of skepticism will negatively affect adoption of the technology while maximized trust will create blind faith in outcomes that are subject to error (45). Promissory discourses like those found on sampled start-up websites that characterize voice AI health-tech as more objective, accurate, and accessible than existing screening methods carried out by human doctors may inspire maximized trust in an AI-based technology that is not infallible. Alternatively, if any of these start-ups rush their technology to market before it is reliable and are scandalized in some way, the level of human-AI trust in general, as well as trust in the voice AI health-tech field, would be negatively impacted.

Alongside other authors and institutional actors in the field (46, 47), we argue that transparency should be the basis for producing trustworthy discourse in the voice AI health-tech sector. One such institutional actor is the World Economic Forum (WEF), which has defined digital trust as “individuals’ expectation that digital technologies and services – and the organizations providing them – will protect all stakeholders’ interests and uphold societal expectations and values” (47). In a white paper, the WEF provides a digital trust framework based on a set of dimensions: cybersecurity, safety, transparency, interoperability, auditability, redressability, fairness, and privacy. Notably, transparency is one of these dimensions. The WEF recommends that organizational leaders go beyond transparency requirements to foster digital trust by reducing the information asymmetry between the organization and its stakeholders/end-users. This digital trust framework and institutional recommendation regarding transparency are examples of guidelines to follow until regulation catches up and is able to the adequately validate voice AI health-tech.

The reliance on promissory descriptors and the lack of transparency around datasets observed in the sampled voice AI health-tech start-up websites should be understood within the structural and economic pressures these companies face, rather than as deliberate attempts to mislead interested parties. Start-ups are fragile actors, navigating a competitive, resource-scarce landscape where their survival depends on their ability to attract investors, differentiate their products, and gain credibility in an uncertain regulatory environment. The norms of digital health marketing—characterized by optimistic, forward-looking claims—are not unique to the voice AI sector but are deeply embedded in the broader innovation ecosystem, where self-promotion is often equated with legitimacy. For many start-ups, adopting this discourse is less a choice than a necessity for competing in a market that demands rapid growth and demonstrable value.

However, this reliance on promissory language is not without consequence. It risks fostering inflated expectations and blind trust in voice AI technologies that are still emerging, and whose reliability and robustness remain subject to significant limitations. While start-ups may not be individually culpable for these patterns, they operate within a system that incentivizes opacity and overstatement to maintain their position. Recognizing this nuance is critical: the problem is not the actions of start-ups alone, but the broader structural dynamics that reward such practices. Therefore, addressing these issues requires systemic solutions—such as standardized transparency protocols, ethical governance frameworks, and marketing guidelines—that shift incentives and foster a more balanced discourse. These measures would enable start-ups to communicate the potential of their technologies responsibly while preserving public trust and ensuring that the voice AI health-tech sector matures in a sustainable and credible manner.

## 6 Limitations

There are several limitations to acknowledge in this study. First, the 25 voice AI health-tech start-ups identified for this study, while comprehensive for this nascent field, is still relatively small. Expanding the analysis to a broader cross-section of companies could uncover additional discursive patterns or nuances missed here; but it could also explain why certain trends are in fact shared by health-tech start-ups and highlight even in greater clarity what is specific to the voice AI sector. Second, the exclusion of non-English websites prevented a more international perspective on how these start-ups operate across cultures. Given language and cultural influences on how technologies are communicated and received, future analyses should endeavor to be multilingual and multinational to fully capture global discursive variation. Third, it is important to point out that the selected sample of start-ups are, with one exception, all from OECD countries in the Global North. The results are thus not generalizable to the context and health tech market in the Global South. A fourth limitation is that this analysis focused only on the textual content of start-up websites, excluding other linked materials such as scientific publications, blog posts, media articles, advertisements, images, graphics, and awards. While websites provide core public messaging, other communications, including investor pitches, social media, and scientific publications, may use different rhetorical strategies not captured here. A multimedia analysis across various message channels could offer complementary insights. Additionally, start-ups may share more detailed information, such as training data, with clinicians or users upon request for a demo.

Fifth, the static nature of this website snapshot that offers just one cross-sectional view. As companies evolve, so too may their public discourses in ways a one-time analysis cannot account for. Longitudinal studies tracking start-up rhetoric over multiple growth stages could elucidate interesting discursive shifts. Relatedly, the context and motivations underlying the identified discursive patterns remain interpretive given the analytical approach's sole reliance on textual data. Interviews or ethnographic studies with start-up founders, employees, and other stakeholders could shed light on the intentionality and drivers behind the language choices.

Finally, it is important to reiterate that discourse analysis as a qualitative methodology does not produce findings generalizable in the same manner as quantitative experimental research. The insights here represent systematically identified patterns and research-derived interpretations, rather than statistically significant facts. As such, the knowledge claims are suggestive and theory-building rather than conclusive. That said, these limitations are common for much exploratory, interpretive research aiming to open up new conceptual framings and lines of inquiry. The goal is to stimulate further investigation, dialogue, and activity around an emerging issue space. The limitations demarcate avenues for subsequent studies building on these initial explorations using diverse methodologies.

## 7 Conclusion

The sampled voice AI health-tech start-up website discourse reveals three patterns: the use of consistent (1) thematic and (2) stylistic descriptors typical of the digital health field and (3) variation (but most often lack of) in the amount of information about training data. We hypothesize that the main function of these patterns is to market this new technology as a legitimate, valuable, transformative digital health product to a diverse set of interested parties, including investors, larger firms, clinicians, healthcare administrators, and end-users.

Voice AI health-tech start-ups are fragile actors dependent on external funding that must appear legitimate to a diverse set of actors, prove the worth of their innovative products, and navigate complex regulations within various healthcare systems. These contextual elements explain the patterns of consistent thematic and stylistic descriptors and varied amounts of information about training data we identified in the sampled website discourse. Start-ups are fragile due to their reliance on investment and external funding, and the healthcare system is a murky regulatory environment for such small private sector actors with limited resources. In order to survive, these start-ups must prove their legitimacy and distinctiveness to both stakeholders and end-users. Promises that start-ups’ technology is cost-effective, accurate, and objective, among other descriptors, is the norm in digital health, and thus a signal of legitimacy. A start-up using more cautious language to describe their product would stand out as weak in a field where self-promotion is the standard discourse.

The discourse on voice and speech datasets is less promissory and often completely neglected in this sample. The current lack of standards and general lack of incentives for the private sector to meet certain standards, or to standardize the way their products are presented and marketed help explain the dearth of discourse on datasets and voice AI disclosure. We argue that transparency about the size and diversity of training datasets would make start-ups more trustworthy to various stakeholders, including health-care providers and patients. Integrating AI-based health-tech into healthcare systems will require more trust and explainability in these technologies, and transparency is a step in the right direction.

To facilitate the maturation of the voice AI health-tech sector, there is a pressing need for standardization in the presentation of data practices, transparency around voice AI products, and their associated benefits and risks. Central to this is the development of a standardized protocol for voice data to ensure consistency, reliability, and fairness in the datasets used to train these technologies. Additionally, establishing international standards for applied model cards—describing how voice AI solutions are implemented or applied in real-world health use cases—can enhance clarity and comparability across products. Marketing ethics guidance is equally essential to ensure that promotional materials accurately reflect the capabilities, limitations, and evidence supporting these technologies. Together, these frameworks would foster trust, accountability, and alignment with regulatory and ethical expectations. Start-ups are crucial actors in the voice AI health-tech field as they are the first to be developing products that implement this promising technology and integrate it into clinical settings. Collaboration between start-ups and researchers in the public sector can facilitate the development and implementation of standards and protocols that could improve transparency in the private sector of the field.

Discourse analysis suggests that the voice AI health-tech market remains in its formative phase, as it continues to define its identity, establish its value proposition, and justify its role within healthcare systems. By prioritizing transparency, ethical governance, and standardized practices, this sector can move beyond self-promotional discourse, address concerns around reliability and safety, and navigate the complexities of healthcare regulation. These steps will mark significant progress toward long-term legitimacy, trustworthiness, and sustainability, ensuring voice AI health-tech is positioned as a credible, transformative force in healthcare.

## Data Availability

The original contributions presented in the study are included in the article/Supplementary Material, further inquiries can be directed to the corresponding author.

## Footnotes

^1^Start-ups have been de-identified in this study and are quoted and referred to using numbers in the results section to emphasize the collective discourse of the start-up sector of the voice AI health-tech field rather than any start-up's individual statements or claims.

## Group members of Bridge2AI-Voice Consortium

Yael E. Bensoussan, Olivier Elemento, Jean-Christophe Bélisle-Pipon, David Dorr, Satrajit Ghosh, Alistair Johnson, Phillip Payne, Maria E. Powell, Anaïs Rameau, Vardit Ravitsky, Alexandros Sigaras.
